# Genome-wide copy number variations as molecular diagnostic tool for cutaneous intermediate melanocytic lesions: a systematic review and individual patient data meta-analysis

**DOI:** 10.1007/s00428-021-03095-5

**Published:** 2021-04-13

**Authors:** Chiel F. Ebbelaar, Anne M. L. Jansen, Lourens T. Bloem, Willeke A. M. Blokx

**Affiliations:** 1 Department of Pathology, Division of Laboratories, Pharmacy and Biomedical Genetics, University Medical Center Utrecht, P.O. Box 85500, 3508 Utrecht, GA Netherlands; 2 Division of Pharmacoepidemiology and Clinical Pharmacology, Utrecht Institute for Pharmaceutical Sciences, Utrecht University, Utrecht, Netherlands

**Keywords:** Melanocytic, Melanocytoma, Ambiguous, Intermediate, Copy number variation, Meta-analysis

## Abstract

Cutaneous intermediate melanocytic neoplasms with ambiguous histopathological features are diagnostically challenging. Ancillary cytogenetic techniques to detect genome-wide copy number variations (CNVs) might provide a valuable tool to allow accurate classification as benign (nevus) or malignant (melanoma). However, the CNV cut-off value to distinguish intermediate lesions from melanoma is not well defined. We performed a systematic review and individual patient data meta-analysis to evaluate the use of CNVs to classify intermediate melanocytic lesions. A total of 31 studies and 431 individual lesions were included. The CNV number in intermediate lesions (median 1, interquartile range [IQR] 0–2) was significantly higher (*p*<0.001) compared to that in benign lesions (median 0, IQR 0–1) and lower (*p*<0.001) compared to that in malignant lesions (median 6, IQR 4–11). The CNV number displayed excellent ability to differentiate between intermediate and malignant lesions (0.90, 95% CI 0.86–0.94, *p*<0.001). Two CNV cut-off points demonstrated a sensitivity and specificity higher than 80%. A cut-off of ≥3 CNVs corresponded to 85% sensitivity and 84% specificity, and a cut-off of ≥4 CNVs corresponded to 81% sensitivity and 91% specificity, respectively. This individual patient data meta-analysis provides a comprehensive overview of CNVs in cutaneous intermediate melanocytic lesions, based on the largest pooled cohort of ambiguous melanocytic neoplasms to date. Our meta-analysis suggests that a cut-off of ≥3 CNVs might represent the optimal trade-off between sensitivity and specificity in clinical practice to differentiate intermediate lesions from melanoma.

**Supplementary Information:**

The online version contains supplementary material available at 10.1007/s00428-021-03095-5.

## Introduction

Cutaneous melanocytic neoplasms include various tumor types with clinical behavior ranging from indolent to invasive [1]. Histopathologic evaluation is usually sufficient for classification as either benign (nevus) or malignant (melanoma). However, a minority displays ambiguous histopathological features, not allowing definite classification. Studies of preneoplastic melanocytic lesions have shown that intermediate stages exist in the progression from nevus to melanoma, associated with the acquisition of pathogenic genomic aberrations [2, 3]. These observations challenge the notion that melanocytic neoplasms can only be benign or malignant. Therefore, one of the significant changes in the recently updated World Health Organization (WHO) classification of skin tumors is the classification of melanocytic tumors in nine pathways with four-step progression models [4]. As such, the group of intermediate tumors has expanded, for which the term “melanocytoma” has been proposed with two different grades. These lesions present a diagnostic challenge even for expert dermatopathologists [5, 6]. Importantly, incorrect classification might result in either preventable disease progression or substantial unnecessary costs, psychological stress, and additional surgery. Therefore, various ancillary cytogenetic techniques are employed to help distinguish nevi from melanomas, based on the fact that melanomas usually harbor copy number variations (CNVs) whereas nevi do not (or show specific isolated abnormalities) [7]. Cytogenetic techniques such as comparative genomic hybridization (CGH) array and single-nucleotide polymorphism (SNP) array can detect CNVs genome-wide, resulting in improved diagnostic accuracy in ambiguous melanocytic lesions compared to FISH [8, 9]. Thus, CNVs might provide a valuable tool to allow accurate classification. However, to what extent intermediate lesions carry CNVs has not been well established yet, and a CNV cut-off value to distinguish them from melanoma is not well defined. Therefore, we performed a systematic review and individual patient data meta-analysis to evaluate the use of CNVs to classify intermediate melanocytic lesions.

## Method

### Search and study selection

Embase and PubMed were systematically searched for primary research articles published in English until September 2020, using the terms “ambiguous,” “atypical,” “borderline,” “dysplastic,” “intermediate,” “spitzoid,” “uncertain,” or “unclassified,” paired with major keywords for melanocytic lesions (including melanocytic “lesion,” “tumor,” “proliferation,” “neoplasm,” “nevus,” “nevi,” “melanoma,” “melanocytoma,” “MELTUMP,” “spitz*,” “STUMP”). These results were then overlapped with the MeSH/Emtree terms for DNA copy number variations: “copy number”; “CNA”; “CNV”; “chromosomal aberration, duplication, amplification, deletion, alteration”; “comparative genomic hybridization”; “CGH”; or “SNP array.” After duplicate removal, unique records were screened for eligibility based on title and abstract first and full-text records thereafter by two authors (CE, WB) using Rayyan for systematic reviews [10]. Differences were discussed until consensus was reached or through input from a third author (AJ). Last, backward and forward snowballing of included articles was employed to identify additional articles of interest.

### Eligibility criteria and outcomes of interest

Articles were included when reporting on intermediate cutaneous melanocytic lesions using molecular techniques to identify genome-wide CNVs, such as CGH array and SNP array. Studies using next-generation sequencing (NGS) were included when using panels or computational methods allowing genome-wide copy number calling [11, 12]. Studies using FISH or multiplex ligation-dependent probe amplification (MLPA) were excluded since these techniques do not screen genome-wide for CNVs. Case reports, abstracts, poster presentations, and articles reporting on non-cutaneous melanomas or melanoma cell lines were excluded. The primary outcomes of interest were the number of CNVs and the type of chromosomal aberrations. Secondary outcomes were clinical follow-up, genomic aberrations, and histopathological characteristics.

### Data collection and CNV count

CNVs were identified on individual lesion level. Authors were contacted to obtain individual patient data or additional information if needed. Two authors (CE, AJ) independently performed a CNV count based on the reported chromosomal aberrations using a predefined ruleset. Segmental gains, losses, high-level amplifications, aneuploidy, and polyploidy were each counted as one CNV. Homozygous loss was counted as two CNVs. CNVs considered insignificant in some studies because of their association with generally benign behavior, such as loss of 3p21 (*BAP1* gene) and gain of 11p (*HRAS* gene), were included in the CNV count for uniformity. Chromosomal fusions for which both fusion partners were known were counted as one CNV since they result from one translocation event. Copy-neutral loss of heterozygosity (CN-LOH) was registered separately since it is not accompanied by actual copy number changes. In contrast, chromothripsis can comprise many CNVs but constitutes one tumor event. Therefore, chromothripsis was also registered separately. CNV counts were crosschecked against the reported number of CNVs when available. Ambiguities were resolved via contacting corresponding authors, discussion until consensus, or input from a third author (WB).

### Recategorization and reclassification of lesions

All lesions were reviewed in-depth by two authors (CE, WB) and were recategorized and reclassified according to the 2018 WHO classification of skin tumors. Ambiguous lesions were recategorized as either “benign,” “intermediate,” or “malignant” hierarchically based on (1) provided clinical follow-up, (2) WHO definition, and (3) histopathology and additional case information. Ambiguous or benign lesions with metastatic disease beyond regional lymph nodes during follow-up were recategorized as malignant. Positive sentinel lymph node biopsies were not considered sufficient proof of malignancy since a minority of benign lesions occasionally display such behavior [13]. Per WHO definition, *BAP1*-inactivated nevi (BIN), deep penetrating nevi (DPN), cellular blue nevi (CBN), and congenital nevi with proliferative nodules (CNPN) were recategorized as low-grade intermediate. *BAP1*-inactivated melanocytomas (BIM), deep penetrating melanocytomas (DPM), atypical cellular blue nevi (ACBN), melanocytic tumors of uncertain malignant potential (MELTUMP), and pigmented epithelioid melanocytomas (PEM) were recategorized as high-grade intermediate. Subsequently, all lesions were reclassified according to the nine WHO pathways primarily based on provided genomic data. When distinctive genomic drivers were unavailable, lesions were reclassified based on the evaluation of available histopathology, ancillary tests, and additional case information.

### Statistical analysis

First, we created box plots to describe the data. Although these appeared not normally distributed, we also reported means to allow comparison with previously reported research on CNV counts. Second, we performed Mann-Whitney U tests to determine differences in CNV number between lesion categories and within classifications according to WHO pathway. Third, we created receiver operating characteristic (ROC) curves and calculated the *C*-statistic or area under the ROC curve (AUC). Fourth, sensitivity and specificity were calculated for a range of CNV cut-offs (0–7). As sensitivity analyses, we performed these analyses for two alternative categorizations of the lesions: (1) initially reported category and (2) considering low-grade intermediate lesions (BIN, CBN, CN with proliferative nodules, and DPN) as benign. Furthermore, we evaluated an alternative CNV count, including chromothripsis and CN-LOH. Also, we evaluated CNV count based on CGH data or SNP data only. Last, we evaluated sensitivity and specificity irrespective of CNV count by interpreting microarray data as positive for malignancy in the presence of CNVs suspect for melanoma, such as homozygous loss of 9p21 (*CDKN2A*) and gain of 11q13 (*CCND1*), 8q24 (*MYC*), or 6p25 (*RREB1*). All statistical analyses were performed in SPSS version 26.

## Results

### Study selection

**Fig. 1:**
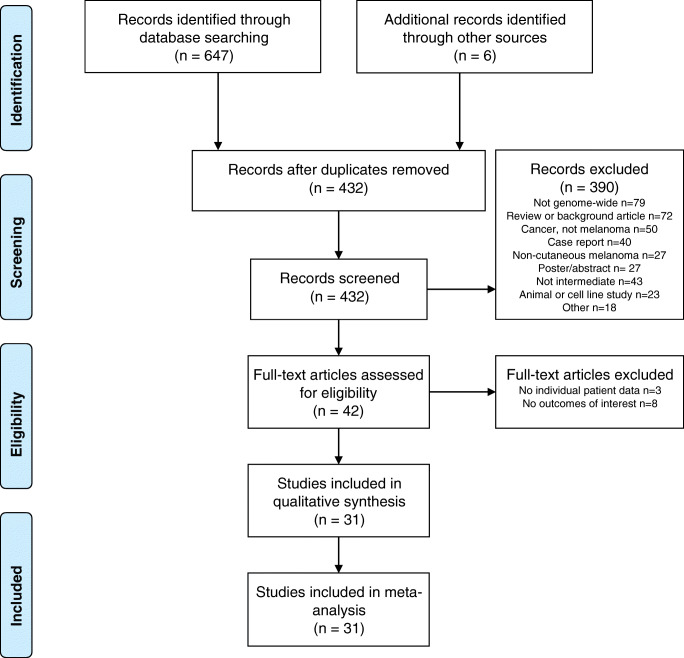
PRISMA flow chart for study selection

Figure 1 shows the PRISMA flowchart for study selection [14]. The search yielded 647 hits, of which 432 were unique records. After assessment for eligibility, 25 studies were included in the meta-analysis, and a further six were identified through snowballing.

### Study characteristics

The characteristics of the 31 included studies are listed in Table 1. All studies were either retrospective (*n*=25), prospective (*n*=4), or mixed (*n*=2) case series. Twenty-two studies used CGH array, six studies used SNP array, and three studies used NGS. In total, data for 431 individual lesions were extracted, of which 252 (58.5%) had been analyzed with CGH array, 144 (33.4%) with SNP array, and 35 (8.1%) with NGS.

**Table 1:** Characteristics of included studies

Study ID	Design	Method	N Ambiguous	N Benign	N Malignant	N Included
Ali 2010 [15]	Retrospective case series	CGH array	1	8	1	10
Alomari 2020 [16]	Mixed case series	SNP array	34	21	40	95
Bastian 2002 [7]	Retrospective case series	CGH array	9	13	6	29
Botton 2013 [17]	Retrospective case series	CGH array	4	1	4	9
Busam 2014 [18]	Retrospective case series	CGH array	2	0	0	2
Carter 2019 [19]	Retrospective case series	SNP array	2	0	1	3
Cellier 2017. [20]	Retrospective case series	CGH array	3	0	0	3
Chan 2016 [21]	Retrospective case series	CGH array	12	5	9	26
Cohen 2017 [22]	Retrospective case series	NGS	13	0	0	13
Cohen 2020 [23]	Retrospective case series	NGS	2	0	5	7
Costa 2016 [24]	Retrospective case series	CGH array	0	3	11	14
Fischer 2017 [25]	Retrospective case series	CGH array	4	0	0	4
Gerami 2020 [26]	Retrospective case series	NGS*	7	10	0	17
Hedayat 2017 [27]	Mixed case series	SNP array	5	5	1	11
Held 2013 [28]	Retrospective case series	CGH array	8	15	0	23
Houlier 2019 [29]	Retrospective case series	CGH array	3	1	1	5
Hung 2016 [30]	Prospective case series	CGH array	2	0	1	3
Lezcano 2020 [31]	Retrospective case series	SNP array	1	0	1	2
Macagno 2020 [32]	Retrospective case series	CGH array	2	0	0	2
Magro 2014 [33]	Prospective case series	CGH array	6	0	0	6
Maize 2005 [34]	Retrospective case series	CGH array	11	11	7	29
Raskin 2011 [35]	Prospective case series	CGH array	16	8	3	27
Redon 2017 [36]	Retrospective case series	CGH array	0	5	5	10
Reimann 2018 [37]	Prospective case series	CGH array	3	0	2	5
Wiesner 2012 [38]	Retrospective case series	CGH array	4	0	0	4
Yeh 2014 [39]	Retrospective case series	CGH array	17	0	0	17
Yeh 2015 [40]	Prospective case series	CGH array	16	0	3	19
Yeh 2016 [41]	Retrospective case series	CGH array	6	2	0	8
Yeh 2019 [42]	Retrospective case series	CGH array	11	5	3	19
Yeh-Botton 2015 [43]	Retrospective case series	CGH array	4	0	2	6
Yelamos 2015 [44]	Retrospective case series	CGH array	4	0	0	4
TOTAL			212	113	106	431

Study characteristics and number of included ambiguous, benign, and malignant lesions per study. *SNP array was used in two cases. *CGH* comparative genomic hybridization, *SNP* single nucleotide polymorphism, *NGS* next-generation sequencing

### Recategorization and reclassification of lesions

Initially, 113 lesions (26.2%) were presented as benign, 212 (49.2%) as ambiguous, and 106 (24.6%) as malignant. Clinical follow-up was available for 297 lesions (68.9%), of which 140 were ambiguous. Two benign and ten ambiguous lesions were recategorized as malignant based on follow-up with distant metastasis or additional case information. Per WHO-definition, 80 lesions were recategorized as low-grade intermediate (28 BIN, 22 CBN, 15 CNPN, and 15 DPN) and 83 lesions as high-grade intermediate (30 ACBN, 7 BIM, 13 DPM, 16 MELTUMP, and 17 PEM). A total of 81 intermediate lesions could not be specified as either low- or high-grade (76 AST, three melanocytomas with *CRTC1*-*TRIM11* fusions, and two melanocytomas with *NRAS* p.Q61R and *IDH1* p.R132C mutations). After recategorization, 69 (16.0%) benign, 244 (56.6%) intermediate, and 118 (27.4%) malignant lesions were available for meta-analysis. Distinctive genomic drivers, including *ALK*, *ROS1*, *NTRK*, *BRAF*, or *MET* fusions and mutational status for *BAP1*, *BRAF, GNA11*, *GNAQ*, *HRAS*, and *NRAS*, were available for 206/431 (47.8%) lesions and 145/244 (59.4%) intermediate lesions. Accordingly, 61/431 (14.1%) lesions were reclassified, mostly “Spitz” lesions carrying a *BRAF* p.V600E or *NRAS* p.Q61R mutation and lesions designated “DPN,” “DPM,” or “MELTUMP” carrying a *GNAQ* p.Q209L or *GNA11* p.Q209L mutation.

### Chromosomal aberrations in intermediate lesions

In our dataset, 18/69 (26.1%) of benign, 134/244 (54.9%) of intermediate, and 112/118 (94.9%) of malignant lesions displayed ≥1 CNV. Within intermediate lesions, 43/80 (53.8%) of low-grade, 35/83 (42.2%) of high-grade, and 56/81 (69.5%) of intermediate lesions not otherwise specified (NOS) displayed ≥1 CNV. The most frequently encountered CNVs in intermediate lesions are listed in Table 2. Loss of 3p spanning the *BAP1* gene on 3p21 was most commonly found, all but one (ACBN) harbored by *BAP1*-inactivated lesions. The most common gain involved 7q, carried mainly by AST. Chromosomal aberrations known to occur in melanoma [7, 9] frequently were infrequent or absent in intermediate lesions (marked with an asterisk in Table 2). Two AST displayed heterozygous loss of 9p21 spanning the *CDKN2A* gene. One ACBN showed a gain of 8q24 spanning the *MYC* gene. Aneuploidies were mainly found in BIN/BIM carrying a loss of chromosome 3 and CNPN carrying a loss of chromosome 7 and gain of chromosome 8. Chromothripsis was found in one malignant Spitz tumor (MST) and five intermediate lesions (two AST, one CBN, one CNPN, and one MELTUMP). Of these, clinical follow-up was only available for the CBN and CNPN. The CBN harbored chromothripsis of chromosomes 3 and 7 and 14 additional CNVs, without evidence of disease during a follow-up of 3.8 years. The CNPN harbored chromothripsis of 1p and two additional CNVs, and the patient was disease-free at 3.5 years after excision. CN-LOH was found in eight melanomas and three intermediate lesions (one DPM, one MELTUMP, and one PEM). The DPM carried CN-LOH of 17q12-qter and did not harbor additional CNVs. The MELTUMP carried CN-LOH of chromosome 7 and had 15 additional CNVs. Clinical follow-up for these cases was not available. The PEM carried CN-LOH of the distal part of chromosome 17q and did not harbor any additional CNVs. Short-term clinical follow-up (not specified) did not show any sign of disease.

**Table 2:** Most frequently found chromosomal aberrations in intermediate melanocytic lesions

Losses	Total (*n*=244)	Low-grade (*n*=83)	High-grade (*n*=83)	Intermediate NOS (*n*=81)	Gains	Total (*n*=244)	Low-grade (*n*=83)	High-grade (*n*=83)	Intermediate NOS (*n*=81)
3p	10.2%	20.0%	9.6%	1.2%	7q*	4.1%	2.5%	1.2%	8.6%
1p	5.3%	0.0%	6.0%	9.9%	2p	2.9%	1.3%	0.0%	7.4%
Entire 3	5.3%	13.8%	2.4%	0.0%	1p	2.0%	0.0%	2.4%	3.7%
2p	3.3%	1.3%	2.4%	6.2%	Entire 8	2.0%	5.0%	1.2%	0.0%
6q*	3.3%	2.5%	3.6%	3.7%	15q	2.0%	0.0%	6.0%	0.0%
Entire 9	3.3%	2.5%	4.8%	2.5%	Entire 20	2.0%	3.8%	1.2%	1.2%
9p*	2.5%	0.0%	1.2%	6.2%	Entire 7	1.6%	1.3%	1.2%	2.5%
7q	2.5%	1.3%	3.6%	2.5%	20p	1.6%	0.0%	4.8%	0.0%
15q	2.5%	0.0%	1.2%	6.2%	1q*	1.2%	0.0%	2.4%	1.2%
3q	2.0%	0.0%	4.8%	1.2%	6p*	1.2%	1.3%	2.4%	0.0%
2q	1.6%	1.3%	0.0%	3.7%	9q	1.2%	0.0%	3.6%	0.0%
6p	1.6%	1.3%	0.0%	3.7%	10p	1.2%	0.0%	0.0%	3.7%
Entire 7	1.6%	5.0%	0.0%	0.0%	Entire 15	1.2%	2.5%	0.0%	1.2%

Most frequent gains and losses found in intermediate melanocytic lesions. Chromosomal aberrations are divided into gain or loss of entire chromosomes and segmental chromosomal aberrations (including focal aberrations and gain or loss of chromosomal arms). Chromosomal aberrations marked with an asterisk represent gains and losses frequently found in melanoma

### CNV counts after recategorization and reclassification

**Fig. 2:**
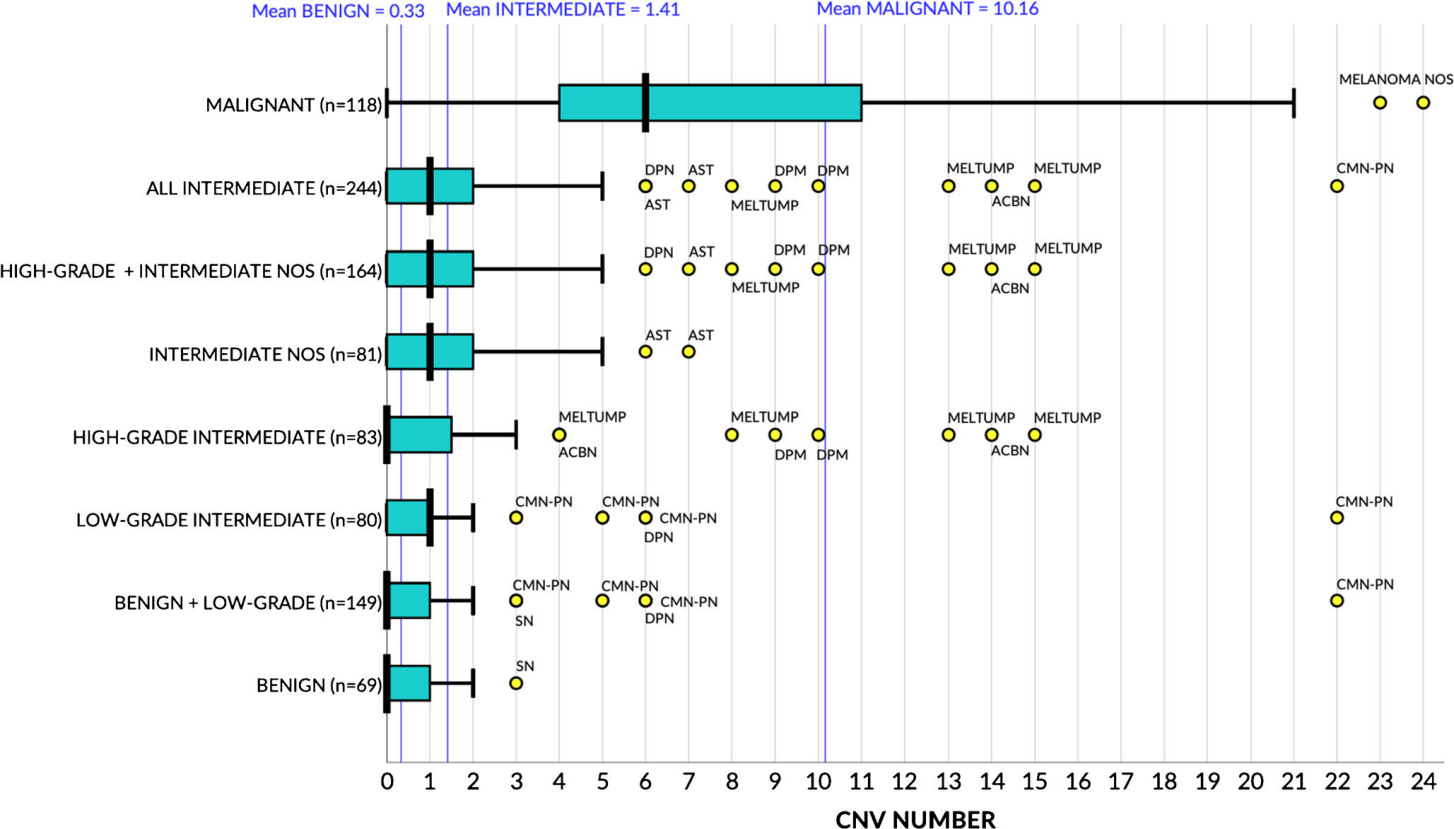
Box and whisker plots showing CNV count after recategorization. Bold vertical black lines show the medians. The blue boxes show the interquartile range (first to third quartile), and the whiskers indicate the maximal value within 1.5 times the interquartile range. The vertical blue lines show the means of all benign, intermediate, and malignant lesions, and the yellow dots represent outliers. NB. Outliers in the malignant category with CNV counts >24 are not shown for clarity of the graph. ACBN, atypical cellular blue nevus; AST, atypical Spitz tumor; CMN-PN, congenital melanocytic nevus with proliferative nodule; DPN, deep penetrating nevus; DPM, deep penetrating melanocytoma; MELTUMP, melanocytic tumor of uncertain malignant potential; SN, Spitz nevus; Melanoma NOS, not otherwise specified

**Fig. 3:**
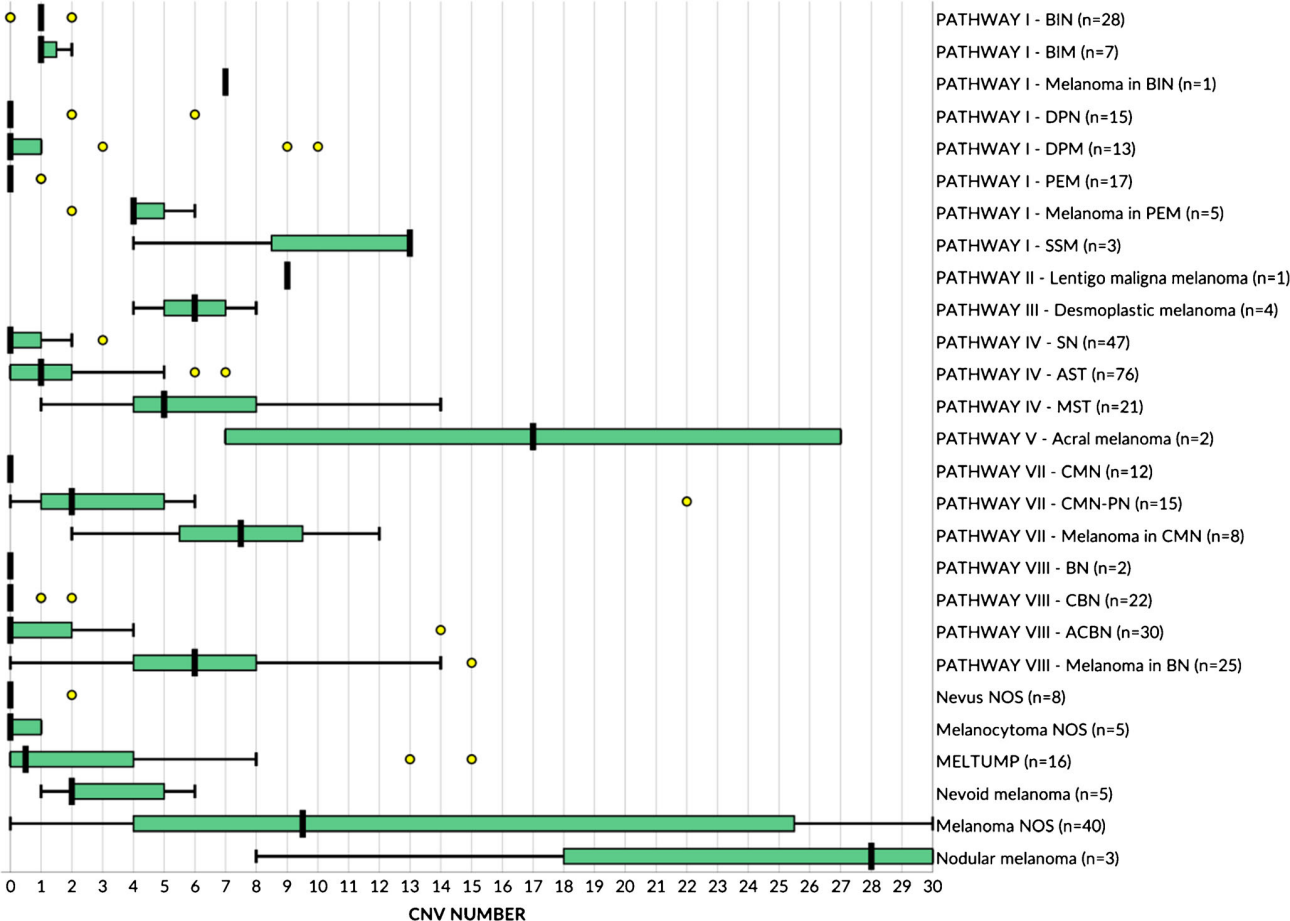
Box and whisker plots showing CNV count after WHO reclassification. Bold vertical black lines show the medians. The green boxes show the interquartile range (first to third quartile), and the whiskers indicate the maximal value within 1.5 times the interquartile range. The yellow dots represent outliers. NB. CNV counts >30 are not shown for clarity of the graph. ACBN, atypical cellular blue nevus; AST, atypical Spitz tumor; BIN, BAP1-inactivated nevus; BIM, BAP1-inactivated melanocytoma; BN, blue nevus; CBN, cellular blue nevus; CMN, congenital melanocytic nevus; CMN-PN, congenital melanocytic nevus with proliferative nodule; DPN, deep penetrating nevus; DPM, deep penetrating melanocytoma; MELTUMP, melanocytic tumor of uncertain malignant potential; PEM, pigmented epithelioid melanocytoma; MST, malignant Spitz tumor; NOS, not otherwise specified; SN, Spitz nevus; SSM, superficial spreading melanoma

Figures 2 and 3 show the number of CNVs per lesion category and WHO class, respectively. The CNV number in intermediate lesions (median 1, interquartile range [IQR] 0–2) was significantly higher (*p*<0.001) compared to that in benign lesions (median 0, IQR 0–1) and significantly lower (*p*<0.001) compared to that in malignant lesions (median 6, IQR 4–11) (Fig. 2). There was no significant difference between low-grade or high-grade intermediate lesions (*p*=0.499). In WHO pathway I, CNV number in BIM (median 1, IQR 1–1.5) was not significantly higher (*p*=0.092) compared to that in BIN (median 1, IQR 1–1) and not significantly higher (*p*=0.449) in DPM (median 0, IQR 0–1) compared to that in DPN (median 0, IQR 0–0). CNV number in PEM (median 0, IQR 0–0) was significantly lower (*p*<0.001) compared to melanomas in PEM (median 4, IQR 4–5). In pathway IV, CNV number in AST (median 1, IQR 0–2) was significantly higher (*p*<0.001) compared to that in Spitz nevi (median 0, IQR 0–1) and significantly lower (*p*=0.001) compared to that in MSTs (median 5, IQR 4–8). In pathway VII, CNV number in CNPN (median 2, IQR 1–5) was significantly higher (*p*<0.001) compared to that in CN (median 0, IQR 0–0) and significantly lower (*p*=0.009) compared to melanomas in CN (median 7.5, IQR 5.5–9.5). Last, in pathway VIII, CNV number in CBN (median 0, IQR 0–0) was not significantly different (*p*=0.585) from blue nevi (median 0, IQR 0–0) but significantly lower (*p*=0.015) compared to that in ACBN (median 0, IQR 0–2). CNV number in ACBN was significantly lower (*p*<0.001) compared to melanomas in blue nevi (median 6, IQR 4–8) (Fig. 3). Relevant outliers in the intermediate category are shown as yellow dots in Fig. 2. The most extreme outlier corresponded to a CNPN harboring 22 CNVs, all gains and losses of whole chromosomes. Clinical follow-up was not available for the MELTUMP with 15 CNVs, the DPM with 10 CNVs, and the AST with 7 CNVs. The remaining lesions did not show evidence of disease during the available relatively short follow-up (varying from 14 to 46 months).

### CNV cut-off value

**Fig. 4:**
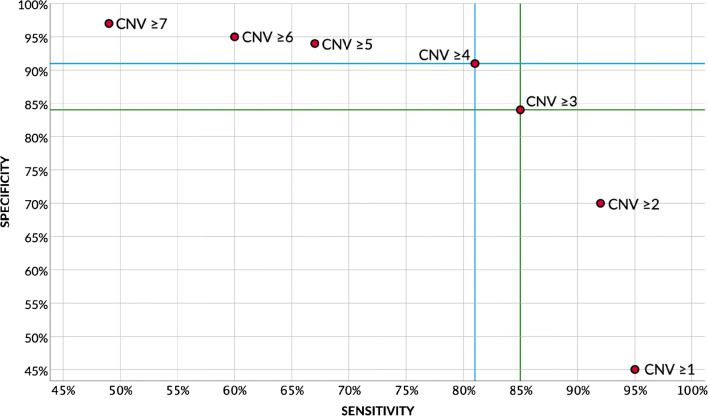
Plot showing the trade-off between sensitivity (x-axis) and specificity (y-axis) to differentiate between intermediate and malignant lesions using different CNV cut-offs. Blue and green lines indicate sensitivity and specificity corresponding to CNV cut-offs of ≥4 and ≥3, respectively. CNV, copy number variation

The *C*-statistic to differentiate between nevi and melanoma was 0.96 (95% CI 0.93–0.99, *p*<0.001) and between intermediate lesions and melanoma 0.90 (95% CI 0.86–0.94, *p*<0.001), indicating excellent ability to differentiate using CNV number [45]. In contrast, the CNV number displayed poor ability to differentiate between intermediate and benign lesions (*C*-statistic 0.67, 95% CI 0.61–0.73, *p*<0.001). Figure 4 shows sensitivity and specificity for differentiating intermediate from malignant lesions given various CNV cut-off values. Using a cut-off of ≥3 CNVs, 85% of malignant lesions would be correctly categorized as malignant (sensitivity), and 84% of non-malignant lesions would be correctly classified as non-malignant (specificity).

### Sensitivity analyses

None of the alternative lesion categorizations or alternative CNV counts substantially changed the results for differentiation between intermediate and malignant lesions. The AUC based on the initially reported category was 0.88 (95% CI 0.84–0.92, *p*<0.001). The AUC when considering low-grade intermediate lesions benign remained 0.90 (95% CI 0.86–0.94, *p*<0.001). The AUC, when including chromothripsis and CN-LOH in the CNV count, also remained 0.90 (95% CI 0.86–0.94, *p*<0.001). The AUC based on SNP array or CGH data only decreased to 0.85 (95% CI 0.78–0.92, *p*<0.001) and increased to 0.94 (95% CI 0.91–0.97, *p*<0.001), respectively. Including specific CNVs suspect for melanoma as a positive test marker did not substantially change these results.

### Risk of bias across studies

The risk of bias was generally considered low to unknown and constituted mainly selection and information bias. Most studies used archival cases without adequately defining the selection process, creating an unknown selection bias risk. Three studies reported CNVs for selected representative cases. Comprising only 10 cases, we consider the impact of potential selection bias very low. In addition, the detection of CNVs is highly dependent on the type of microarray, resolution, DNA quality, and sample purity. Most studies used archival DNA from formalin-fixed paraffin-embedded (FFPE) tissue and did not report tumor cell percentages, which introduces an unknown risk of information bias.

## Discussion

To the best of our knowledge, this is the first systematic review and individual patient data meta-analysis to assess genome-wide CNVs as a diagnostic tool for intermediate melanocytic lesions, using cytogenetic tests such as SNP array and CGH array.

Chromosomal aberrations were found in 55.1% of intermediate lesions. Gains and losses frequently seen in melanoma, such as gain of 1q, 6p, and 7q and loss of 6q and 9p, were uncommon in intermediate lesions. CN-LOH and chromothripsis were only found in intermediate and malignant lesions. Our analysis shows that the median number of CNVs in intermediate lesions is statistically significantly higher compared to that in nevi and lower compared to that in melanoma. Similarly, the number of CNVs significantly increased in WHO pathway IV (Spitz), VII (congenital), and VIII (blue) along the spectrum from nevus to melanoma. In contrast, the CNV number was not statistically different between BIN and BIM and between DPN and DPM. Surprisingly, “high-grade” melanocytomas (ACBN, BIM, DPM, PEM, and MELTUMP) carried CNVs less frequently than “low-grade” melanocytomas (BIN, CBN, CMN with proliferative nodules, and DPN). This observation demonstrates the difficulty of grading intermediate lesions using a four-tier system as is used in the WHO classification. Yet, our results suggest CNVs demonstrate excellent ability to differentiate between intermediate melanocytic lesions and melanoma in clinical practice. A cut-off of ≥3 CNVs corresponded to 85% sensitivity and 84% specificity, and a cut-off of ≥4 CNVs corresponded to 81% sensitivity and 91% specificity, respectively.

Several CNV cut-offs for malignancy have previously been suggested. Based on their case series, Maize et al. and Chan et al. suggested a cut-off of ≥3 CNVs and ≥4 CNVs, respectively, and Alomari et al. proposed an algorithm using ≥4 significant CNVs with additional caveats in case of ≤3 CNVs [16, 21, 34]. Our current meta-analysis integrates and expands their data, providing more robust evidence for various cut-offs in the classification of intermediate lesions. Both a cut-off of ≥3 and ≥4 CNVs can be considered, the first having a higher sensitivity (fewer false-negative diagnoses) and the latter having a higher specificity (fewer false-positive diagnoses). Yet, sensitivity might prevail in clinical practice given the potentially disastrous consequences of a false-negative misdiagnosis, even at the cost of a modestly lower specificity and resulting treatment burden. Therefore, we propose a cut-off of ≥3 CNVs as indicative of malignancy.

Of note, a minority of melanomas did not harbor CNVs, and benign lesions might carry CNVs with limited prognostic value. In contrast, specific CNVs may also be relevant if present in isolation, such as homozygous loss of 9p21 (*CDKN2A*) [16]. Therefore, CNVs should always be interpreted considering the clinicopathological context. Yet, the contextual interpretation of specific CNVs is difficult in unclassified lesions. For example, loss of 3p21 (*BAP1*) is insignificant in BIN/BIM but is of major significance in an ACBN or MEBN. It is currently mostly unknown which CNVs are most predictive for malignancy in the various WHO pathways, and this requires additional research.

The main strengths of this study include the following. All lesions have been vigorously reviewed based on published and unpublished individual patient data using the 2018 WHO classification of skin tumors. As such, our meta-analysis integrates the most recent clinicopathological and genomic insights to establish CNVs in intermediate lesions. Our analyses provide a better-defined CNV cut-off value for malignancy to support clinical decision-making, based on the largest pooled dataset of intermediate lesions to date. The sensitivity analyses strengthened the robustness of the results.

This study has several limitations. First, genome-wide microarray data are difficult to pool since the detection of CNVs is highly dependent on resolution and technical specifications. Second, the detection of CNVs depends on sample quality. Most studies used DNA from archival FFPE blocks and did not report the tumor cell percentages, although the latter should exceed 30% to detect CNVs reliably. Therefore, paucicellular lesions such as PEM and large-cell lesions such as *MAP3K8*-*SVIL* fused ASTs with strong lymphocytic infiltrate render dilution effects and make CNV detection more difficult. In our dataset, a minority of melanomas (5.1%) did not carry any CNVs, and it is unclear if this is due to dilution or truly represents a lack of CNVs. This might have negatively influenced sensitivity, although our ROC analysis still showed excellent discriminative ability. Third, rules for CNV counts are not uniformly defined, and we were only able to count CNVs that were reported or provided by authors. CNVs reported to be attributable to a chromosomal fusion were counted as one CNV. This may have slightly overestimated the CNV number, especially in Spitz tumors, where fusions are a common driver event and probably not relevant for prognosis. Also, a minority of melanomas were reported to harbor extremely high CNV counts (>30), which likely included aberrations not included in our CNV count, such as chromothripsis. However, it is unlikely this substantially affected our results since we performed non-parametric tests. Fourth, clinical outcomes were not available in 34.0% of ambiguous cases. Follow-up with distant metastasis remains the gold standard for proof of malignancy and might still occur years after diagnosis. Yet, clinical follow-up was available for most outliers in the intermediate category. Fifth, distinctive driver mutations were available in 59.4% of intermediate lesions. For the remaining lesions, classification was based on histopathology alone, which is more subjective than genomic data. Last, we established our ROC analysis on one variable (CNV number), whereas ideally, diagnostic evaluation is performed via multivariable analysis, including all available diagnostic information. Nonetheless, it indicated excellent discriminative ability, which supports further research in a dedicated dataset. Despite these limitations, we believe this meta-analysis provides robust results applicable to general dermatopathology practice.

To conclude, this systematic review and individual patient data meta-analysis provides a comprehensive overview of CNVs in cutaneous intermediate melanocytic lesions and a diagnostic interpretation of different CNV cut-offs for malignancy, based on the largest pooled cohort of ambiguous melanocytic neoplasms to date. Our results suggest that a cut-off of ≥3 CNVs might represent the optimal trade-off between sensitivity and specificity in clinical practice to distinguish intermediate from malignant lesions. Future research should externally validate this cut-off in a distinct dataset, assess the predictive value of specific CNVs in the various WHO pathways, and correlate genome-wide microarray data with objective genomic and clinical parameters.

## Supplementary Information

ESM 1 (SAV 791 kb)
